# Encapsulation of Risperidone by Methylated β-Cyclodextrins: Physicochemical and Molecular Modeling Studies

**DOI:** 10.3390/molecules25235694

**Published:** 2020-12-03

**Authors:** Laura Sbârcea, Ionuț-Mihai Tănase, Adriana Ledeți, Denisa Cîrcioban, Gabriela Vlase, Paul Barvinschi, Marinela Miclău, Renata-Maria Văruţ, Cristina Trandafirescu, Ionuț Ledeți

**Affiliations:** 1 Department of Pharmacy I, Faculty of Pharmacy, “Victor Babeş” University of Medicine and Pharmacy, 2 Eftimie Murgu Square, 300041 Timisoara, Romania; sbarcea.laura@umft.ro (L.S.); circioban.denisa@umft.ro (D.C.); ionut.ledeti@umft.ro (I.L.); 2 Advanced Instrumental Screening Center, Faculty of Pharmacy, “Victor Babes” University of Medicine and Pharmacy, 2 Eftimie Murgu Square, 300041 Timisoara, Romania; 3 Faculty of Industrial Chemistry and Environmental Engineering, Politehnica University of Timisoara, 6 Vasile Parvan Blvd, 300223 Timisoara, Romania; ionut.tanase@student.upt.ro; 4 Research Centre for Thermal Analysis in Environmental Problems, West University of Timisoara, 16 Pestalozzi Street, 300115 Timisoara, Romania; gabriela.vlase@e-uvt.ro; 5 Faculty of Physics, West University of Timisoara, 4 Vasile Parvan Blvd, 300223 Timisoara, Romania; pc_barvi@yahoo.fr; 6 National Institute for Research and Development in Electrochemistry and Condensed Matter, 144 Dr. A. Păunescu-Podeanu Street, 300587 Timisoara, Romania; marinela.miclau@gmail.com; 7 Faculty of Pharmacy, University of Medicine and Pharmacy Craiova, 2–4 Petru Rares Street, 200349 Craiova, Romania; rennata_maria@yahoo.com; 8 Department of Pharmacy II, Faculty of Pharmacy, “Victor Babeş” University of Medicine and Pharmacy, 2 Eftimie Murgu Square, 300041 Timisoara, Romania; trandafirescu.cristina@umft.ro

**Keywords:** risperidone, cyclodextrins, molecular encapsulation, inclusion complex, solubility

## Abstract

Risperidone (RSP) is an atypical antipsychotic drug which acts as a potent antagonist of serotonin-2 (5TH2) and dopamine-2 (D2) receptors in the brain; it is used to treat schizophrenia, behavioral and psychological symptoms of dementia and irritability associated with autism. It is a poorly water soluble benzoxazole derivative with high lipophilicity. Supramolecular adducts between drug substance and two methylated β-cyclodextrins, namely heptakis(2,6-di-O-methyl)-β-cyclodextrin (DM-β-CD) and heptakis(2,3,6-tri-O-methyl)-β-cyclodextrin (TM-β-CD) were obtained in order to enhance RSP solubility and improve its biopharmaceutical profile. The inclusion complexes were evaluated by means of thermoanalytical methods (TG—thermogravimetry/DTG—derivative thermogravimetry/HF—heat flow), powder X-ray diffractometry (PXRD), universal-attenuated total reflectance Fourier transform infrared (UATR-FTIR), UV spectroscopy and saturation solubility studies. Job’s method was employed for the determination of the stoichiometry of the inclusion complexes, which was found to be 2:1 for both guest–host systems. Molecular modeling studies were carried out for an in-depth characterization of the interaction between drug substance and cyclodextrins (CDs). The physicochemical properties of the supramolecular systems differ from those of RSP, demonstrating the inclusion complex formation between drug and CDs. The RSP solubility was enhanced as a result of drug encapsulation in the CDs cavity, the higher increase being obtained with DM-β-CD as host; the guest–host system RSP/DM-β-CD can thus be a starting point for further research in developing new formulations containing RSP, with enhanced bioavailability.

## 1. Introduction

Cyclodextrins (CDs) are macrocyclic molecules that have been extensively used in the last decade in the pharmaceutical field, cosmetics, food, textile, separation science and chemical industries. They contain six, seven or eight D-glucopyranose units (α, β and γ-cyclodextrin) linked in α (1–4) to form a hollow, truncated-cone-shape structure with hydrophilic outer surface and hydrophobic internal cavity. This particular structure is responsible for CDs property of establishing specific interaction with a variety of drug molecules; guest–host inclusion complexes are thus formed by trapping the drug into CD cavity [1,2,3,4,5]. Molecular encapsulation of bioactive compounds in the CDs has achieved pharmaceutical relevance since the physical, chemical and biopharmaceutical characteristics of guest molecules are remarkably improved. CD inclusion complexes can increase the solubility of insoluble drug molecules and the antioxidant properties, can improve the chemical stability, the biological activity and the bioavailability of guest substances, may reduce/eliminate the unpleasant smell and taste, may prevent drug–excipient or drug–drug interactions [6,7,8,9,10,11,12,13,14,15]. β-cyclodextrin (β-CD) is the most frequently used in the pharmaceutical field due to its availability, appropriate internal cavity size for an important number of drug substances and economic advantages. β-CD is also non-toxic, biodegradable, but it has a limited aqueous solubility [2,4,16]. In order to enhance its solubility, CD derivatives have been developed, among them methylated β-CDs [17,18]. 

**Figure 1:**
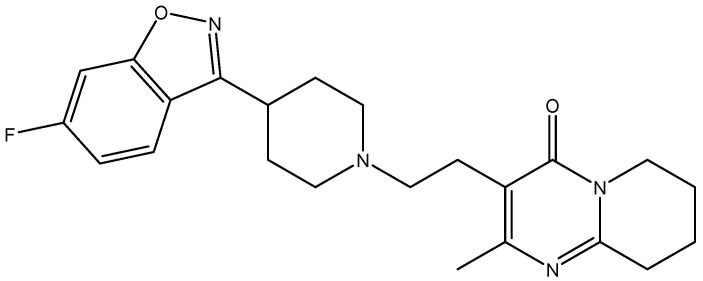
Structural formula of risperidone (RSP).

Risperidone (abbreviated RSP in this paper), *3-[2-[4-(6-fluoro-1,2-benzoxazol-3-yl)piperidin-1-yl]ethyl]-2-methyl-6,7,8,9-tetrahydropyrido [1,2-a]pyrimidin-4-one* (Figure 1) is an atypical antipsychotic drug, belonging to the class of pyridopyrimidines used in treating schizophrenia, behavioral and psychological symptoms of dementia and irritability associated with autism [19,20]. It is a potent inhibitor of serotonin-2 (5TH2) and dopamine-2 (D2) receptors in the brain. RSP belongs to class II in the Biopharmaceutics Classification System (BCS); it is practically insoluble in water and exhibits high lipophilicity (log *P* of 3.49) [19,21,22]. RSP presents the ability to form polymorphs [19], that can possess different solubility, dissolution rates, affecting thus the biopharmaceutical profile of drug substance [23]. 

Among the different strategies aiming at improving the solubility of drug substances with low solubility and high permeability, inclusion complexation with CDs is a valuable approach [23]. Encapsulation of RSP in CDs has been mentioned in the several papers; inclusion complexes of drug with β-CD and hydroxypropyl-β-CD (HP-β-CD) have been investigated and formulated for parenteral administration and nasal application [24,25], solid dispersion of risperidone with methyl-β-cyclodextrin has been studied and incorporated into orally disintegrating tablets for faster release of drug substance [22]. Moreover, the solubility of RSP in aqueous solutions of α-, β-, γ- and HP-β-CDs has been assessed [26].

The major challenge with the design of oral dosage forms lies with their poor bioavailability. Solubility is an essential property of drugs, being one of the most critical and important parameters influencing their bioavailability. Thus, the enhancement of drug solubility and thereby its bioavailability remains one of the most challenging aspects of the drug development, process especially for oral pharmaceutical formulation [27].

In this study we aimed to improve RSP solubility by obtaining the inclusion complexes of drug substance with two methylated cyclodextrins, heptakis(2,6-di-O-methyl)-β-cyclodextrin (DM-β-CD) and heptakis(2,3,6-tri-O-methyl)-β-cyclodextrin (TM-β-CD). UV-spectroscopy, powder X-ray diffractometry (PXRD), thermogravimetry (TG), derivative thermogravimetry (DTG), heat flow (HF) and FTIR spectroscopy were used to evaluate the RSP/CD systems. In addition, molecular modeling studies were carried out for the geometry of complexes investigation. The saturation solubility studies were further performed in order to assess the increase in RSP solubility.

## 2. Results and Discussion

### 2.1. Stoichiometry Determination of RSP/CD Inclusion Complexes

**Figure 2:**
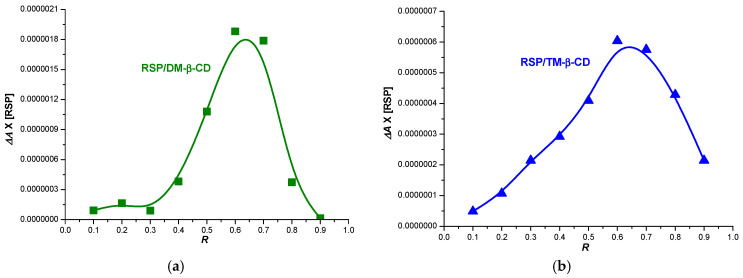
Job’s plot corresponding to RSP/heptakis(2,6-di-O-methyl)-β-cyclodextrin (DM-β-CD) (**a**) and RSP/heptakis(2,3,6-tri-O-methyl)-β-cyclodextrin (TM-β-CD) (**b**) inclusion systems.

Job’s method was used to estimate the stoichiometry of the inclusion complexes [28]. According to this method the inclusion complex stoichiometry can be determined when a measurable physical parameter that correlates linearly with the complex concentration (i.e., absorbance) is plotted against guest mole fraction. The stoichiometry is provided by the value of the molar ratio R that correlates with the maximum concentration of the complex [10,12,29]. The maximum variation (Δ*A*) in RSP absorption is noticed at mole fraction of ~0.66 in the presence of both DM-β-CD and TM-β-CD, which suggests that the main guest–host stoichiometry is 2:1 for both inclusion complexes (Figure 2).

### 2.2. Molecular Modeling Studies

Molecular modeling was used in order to predict the spatial conformation of RSP/CD inclusion complexes in 2:1 ratio and to elucidate the type of bonds involved in the interaction between drug substance and the two CDs. As the expected stoichiometry of RSP:CD inclusion complexes was 2:1, two consecutive docking cycles were carried out starting and selecting the best result from each cycle. 

The molecular docking analysis was performed using the Autodock 4.2.6 software together with the AutoDockTools [30]. The software applies a semi-empirical free energy force field and grid-based docking to assess conformations during docking process. The force field includes six pair-wise evaluations (*V*) and an estimate of the conformational entropy lost upon binding (ΔSconf):

(1) $$\Delta G=\left(V_{bound}^{L-L}-V_{unbound}^{L-L}\right)+\left(V_{bound}^{T-T}-V_{unbound}^{T-T}\right)\;+\;\left(\;V_{bound}^{T-L}-V_{unbound}^{T-L}+\Delta S_{conf}\right)$$

where *L* refers to the “ligand” and *T* refers to the “target” in a ligand–target docking calculation. Each of the pair-wise energetic terms includes evaluations for dispersion/repulsion, hydrogen bonding, electrostatics and desolvation [31]. Following the redocking analysis, we calculated the root-mean-square deviation (RMSD) values to be lower than 0.4 A, suggesting the robustness and repeatability of the docking analysis.

**Figure 3:**
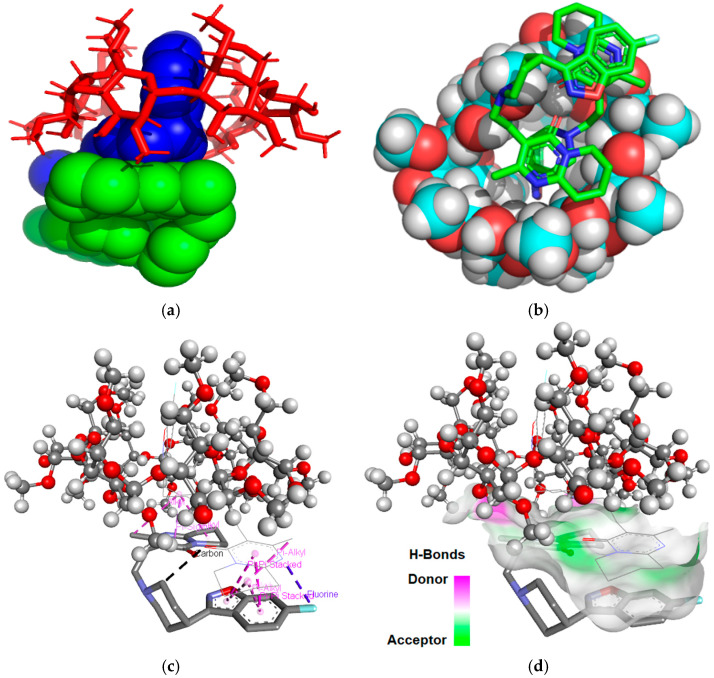
Inclusion complex simulation for a 2:1 molar ratio between RSP and DM-β-CD. Images (**a**), (**b**) show the inclusion complex from the secondary face of DM-β-CD’s cavity. RSP guest molecules are represented in spheres green and blue (**a**), in sticks colored by element (**b**) while DM-β-CD is represented in red sticks (**a**), in sphere colored by element (**b**); image (**c**) shows contacts between RSP and DM-β-CD, RSP is colored by element, DM-β-CD is presented in ball and sticks; image (**d**) show H-bond surface interaction between RSP and DM-β-CD.

**Figure 4:**
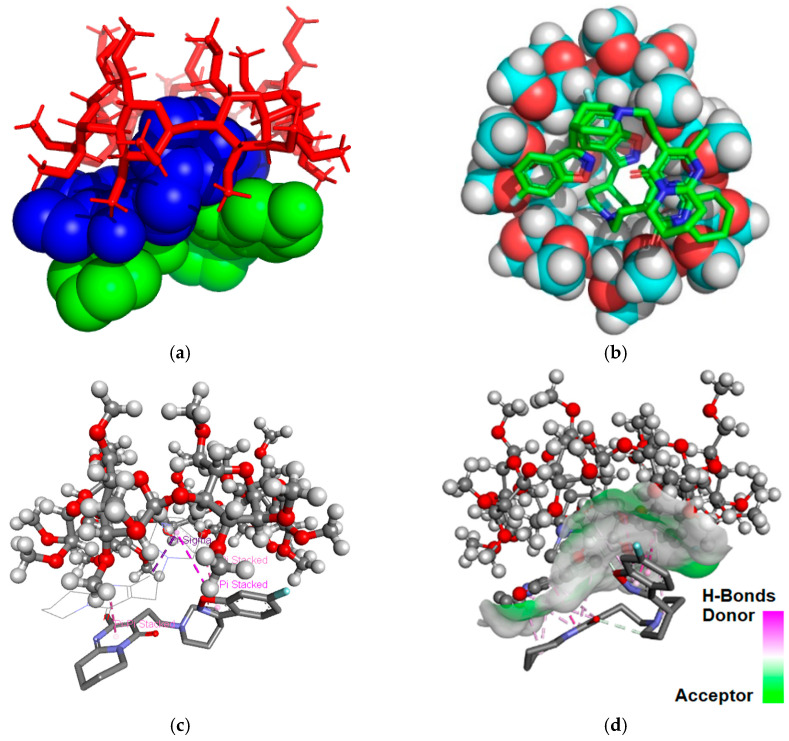
Molecular docking results revealing the geometry of inclusion complex for 2:1 molar ratio between RSP and TM-β-CD. Guest substance is presented in spheres green and blue (**a**), in sticks colored by element (**b**) and host TM-β-CD, in red sticks (**a**), in sphere colored by element (**b**); image (**c**) shows contacts between RSP colored by element and TM-β-CD, in ball and sticks; image (**d**) shows H-bond surface interaction between RSP and TM-β-CD.

The binding free energy values calculated was −12.48 kcal/mol for the RSP/DM-β-CD (2:1) inclusion complex and −13.13 kcal/mol for RSP/TM-β-CD (2:1) inclusion system. According to our data, the RSP/TM-β-CD (2:1) is more stable than the RSP/DM-β-CD (2:1) because it has a lower value of binding free energy. Figure 3 and Figure 4 present the theoretical RSP/CD inclusion complexes, as rendered in the PyMOL [32] and Discovery Studio molecular visualization systems, simulated in 2:1 molar ratio.

In the 3D images of the RSP/DM-β-CD (2:1) interaction we noticed the presence of two Pi-donor non-classical hydrogen bonds, one between fluorine (RSP included in DM-β-CD) and the hydrogen (hydrogen methoxy group) of a glucopyranose unit with a length of 2.37 Å, and second between 1,2-oxazolic heterocyclic oxygen and the hydrogen methoxy group of DM-β-CD (3.04 Å). The benzene ring near the 1,2-oxazolic heterocycle forms Pi–sigma interaction with a length of 2.68 Å with hydrogen methoxy group of carbohydrate moiety (position 6). The fourth non-classical hydrogen bond occurs between the nitrogen group from the 1,2-oxazolic heterocycle and the hydrogen from position 4 of a carbohydrate moiety. The second RSP molecule interacts strongly with the one included in the DM-β-CD cavity, with five Pi–alkyl interactions (4.14 Å, 4.76 Å, 5.40 Å, 4.60 Å and 4.80 Å, respectively) two Pi–Pi stacked interactions (3.68 Å and 4.77 Å) and one halogen bond (Pi–fluorine interaction) with a length of 3.27 Å.

Analyzing the 3D images of the RSP/TM-β-CD (2:1) interaction, we observe that three non-classical hydrogen bonds are formed between the 1,2-oxazole heterocyclic oxygen of RSP included in cavity and the hydrogen (position 4) of three glucopyranose units with a length of 3.65 Å, 3.77 Å and 3.72 Å. Two intramolecular Pi–sigma bonds of 3.35 Å and 3.59 Å, respectively, are established between the 1,2-oxazole heterocycle/benzene ring and the pyrimidin-4-one methyl radical, that leads to conformation changes in the ligand structured included in the cavity. The second RSP molecule not included in the cavity binds to TM-β-CD through a non-classical Pi-donor hydrogen bond between fluorine and the hydrogen of a hydroxyl group (position 2), having a length of 3.52 Å. Moreover, the second molecule of RSP binds to the one included in the cavity by three Pi–Pi stacked interactions with lengths of 3.46 Å, 4.76 Å and 5.76 Å, respectively. A non-classical hydrogen bond with a length of 2.76 Å also appears between the two RSP molecules, involving the oxygen of the 1,2-oxazolic heterocyclic moiety and the carbon atom from position 2 of the piperidine ring.

### 2.3. X-ray Diffraction Studies

**Figure 5:**
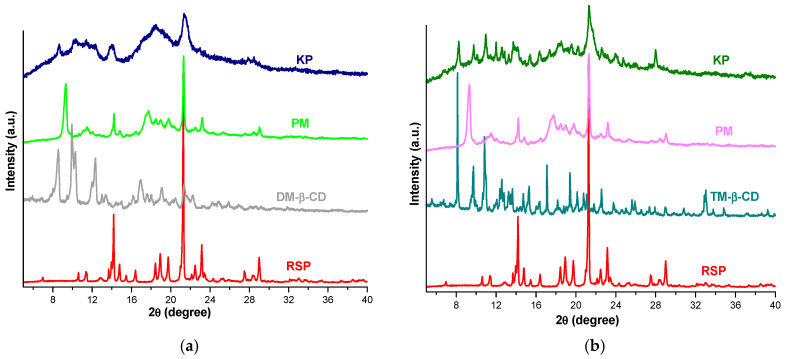
XRD spectra of RSP, DM-β-CD and their PM and KP (**a**); RSP, TM-β-CD, RSP/TM-β-CD PM and KP (**b**).

PXRD was employed in order to characterize the interaction between RSP and CDs in solid state. Figure 5 presents the diffraction patterns of RSP, DM-β-CD, TM-β-CD and RSP/CD binary systems obtained by physical mixture (PM) and by kneading (kneaded products, KP).

The diffractometric profile of RSP reveals two peaks of high intensity at 14.19 and 21.27 2*θ* and other characteristic peaks at 14.79; 18.44; 18.93; 19.74; 23.15 and 29.00 2*θ*, indicating the crystalline nature of the drug substance. The diffraction pattern of DM-β-CD also shows a typical crystalline structure with sharp peaks at 8.54; 9.95; 10.29; 12.31; 16.89; 19.04 and 21.32 2*θ* [11]. In the diffractogram of RSP/DM-β-CD PM the disappearance of CD peaks from 8.54; 9.95; 10.29 and 12.31 2*θ* and the presence of a new peak at 9.38 2*θ* can be noticed. Furthermore, the lower intensity of characteristic diffraction peaks of both guest and host substances can be observed. Figure 5a shows a different diffractometric profile of RSP/DM-β-CD KP from those of parent compounds, with main peaks at 8.65; 14.05 and 21.35 2*θ*. In addition, most of the characteristic diffraction peaks of RSP and CD disappeared highlighting an amorphization process of the drug substance in the KP, which is expected to improve RSP solubility and also demonstrates the existence of an interaction between RSP and DM-β-CD [33].

The derivative TM-β-CD shows a crystalline nature (Figure 5b) confirmed by the presence of sharp peaks at 8.14; 9.73; 10.84; 12.58; 15.31; 17.09; 19.40; 22.57 2*θ* [10] in its PXRD spectrum. The RSP/TM-β-CD PM diffraction pattern exhibits the disappearance of CD peaks at 8.14; 9.73 and 10.84 2*θ* along with a diminution of the characteristic crystalline reflections of RSP and CD, indicating that the new compound is less crystalline than the drug and TM-β-CD alone. An important reduction in crystallinity is noticed in the diffractometric profile of RSP/TM-β-CD KP together with the appearance of new characteristic reflections (i.e., at 11.99; 12.88; 13.71 and 27.98 2*θ*) which provides evidence that a new compound is obtained as a result of inclusion complex formation between drug substance and TM-β-CD [34].

### 2.4. Thermal Metods

**Figure 6:**
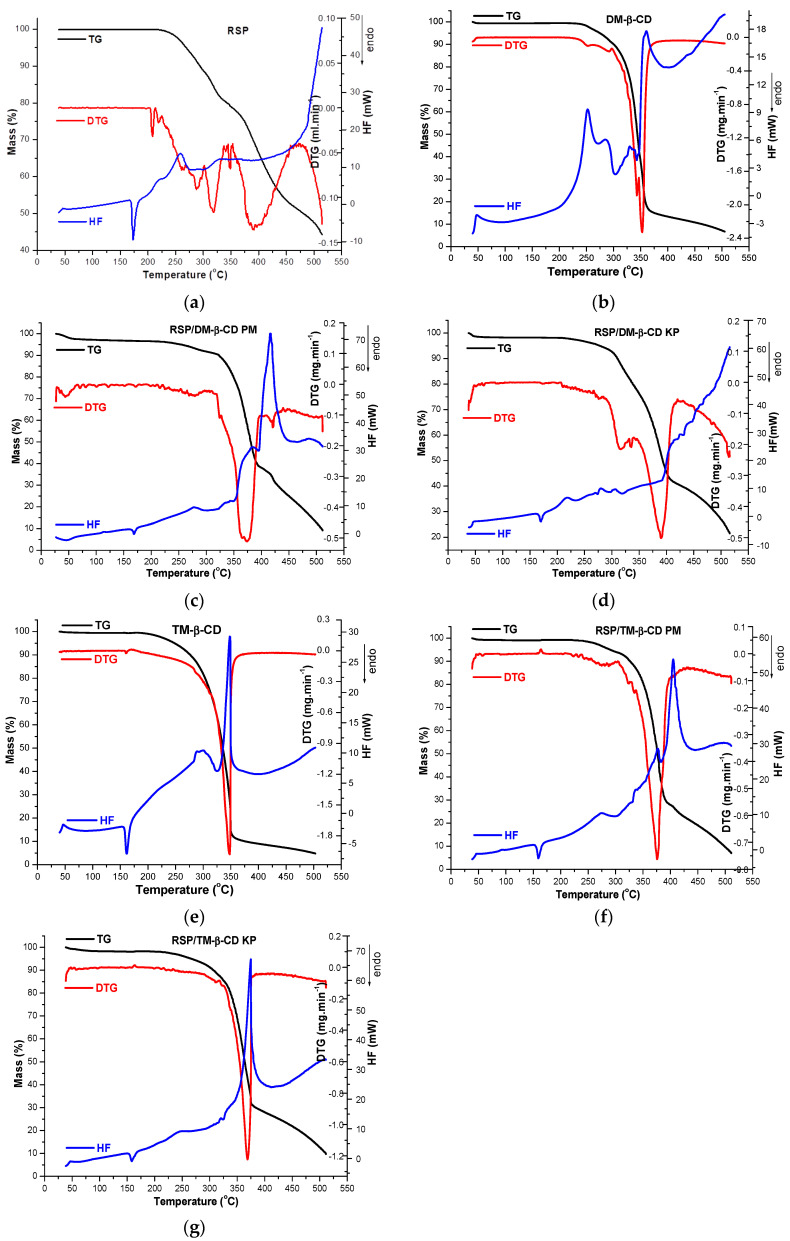
Thermoanalytical curves (thermogravimetry (TG)/derivative thermogravimetry (DTG)/heat flow (HF)) of: RSP (**a**); DM-β-CD (**b**); RSP/DM-β-CD physical mixture (PM) (**c**); RSP/DM-β-CD kneaded products (KP) (**d**); TM-β-CD (**e**); RSP/TM-β-CD PM (**f**) and RSP/TM-β-CD KP (**g**) in air atmosphere, 40–500 °C temperature range and heating rate of 10 °C·min^−1^.

The thermoanalytical curves (TG/DTG/HF) for RSP, DM-β-CD, TM-β-CD, physical mixtures of drug substance and CDs and RSP/CD KP, respectively, are shown in Figure 6a–g.

The TG curve of RSP indicates that the drug substance has a considerable high thermal stability, since no mass loss occurs up to 206 °C. At temperatures higher than 206 °C, a continuous mass loss process is highlighted up to 515 °C (Δ*m* = 55.6%), as RSP suffers molecular breakdowns due to thermal-induced thermooxidations. The decomposition pathway of RSP is complex, this fact being sustained by the DTG curve, where three distinct regions can noticed, as follows: the first one between 200 and 226 °C (peak at 209 °C; Δ*m* = 0.3%), the second, in the temperature range of 226–350 °C, (main peak at 319 °C; Δ*m* = 20.73%) and the last one between 350 and 474 °C (peak at 391 °C, Δ*m* = 28.2%). At temperatures higher than 474 °C, the degradation occurs rapidly, up to 515 °C. The HF curve of RSP reveals an endothermic peak at 173 °C corresponding to the RSP melting [25,35] and a small gradual exotherm event with *T_peak_* = 259 °C assigned to the first process of drug thermal degradation (Figure 6a). A considerable exothermal effect appears above 474 °C, accompanying the rapid mass loss on the TG curve. 

The thermal profile of DM-β-CD (Figure 6b) is relatively simplistic and reveals a good thermal stability of this cyclodextrin up to 232 °C, when the decomposition process begins, showing DTG_peaks_ at 343 and 353 °C, respectively. The HF curve of DM-β-CD shows exothermic effects at 252, 286 and 360 °C attributed to the thermooxidation processes of CD, its melting being probably accompanied and overlapped with these thermooxidations [11,36]. At 500 °C, the solid char has a residual mass 6.73%, so that the degradation of this functionalized polysaccharide is almost complete at this temperature.

Significant changes are observed in the thermal curves of RSP/DM-β-CD binary products when compared with those of the parent compounds. Thus, the RSP endothermic melting peak exhibits a reduction in its intensity and is shifted to lower temperature in HF curves of both PM (*T_peak_* = 169 °C) and KP (*T_peak_* = 170 °C). The RSP exothermic event is displaced to lower temperature in the HF curve of KP (*T_peak_* = 219 °C), while in the PM curve an exothermic peak appears at higher temperature, i.e., 278 °C. Furthermore, the exothermic event in the cyclodextrin HF curve from 360 °C appears in the thermal curve of RSP/DM-β-CD PM at 416 °C (Figure 6c), showing significant reduction in the peak and it is not any more present in HF curve of RSP/DM-β-CD KP (Figure 6d). 

The HF curve of TM-β-CD shows an endothermic event with a maximum at 161 °C assigned to the melting of the substance (Figure 6e). CD presents a good thermal stability up to 190 °C, when the main decomposition process starts, as the TG/DTG curves show (DTG_max_ = 348 °C); an exothermic event with a maximum at 348 °C seen in the HF curve of CD accompanies this process [10].

Notable differences are also observed in the thermal profile of binary systems RSP/TM-β-CD PM and KP (Figure 6f,g). HF curves of PM and KP binary compounds reveal endothermic peaks at 159 °C and 158 °C, respectively, which correspond to the CD melting; they show significant reduction and are slightly shifted to lower temperature in comparison with the pure substance (161 °C). The endothermic peak associated with RSP melting is no longer visible in the curves of both PM and KP; the TM-β-CD exothermic peak is displaced to higher temperature in the curve of PM ((*T_peak_* = 405 °C) and KP (*T_peak_* = 374 °C).

Thermoanalytical methods are among of the most used techniques to characterize the encapsulation of drug substances in the CD cavity and to prove the inclusion complex formation. The thermal profiles of guest molecules that are entrapped in the CD cavity are generally changed [33,34,37,38]. The disappearance of the melting process of RSP from the HF curve of RSP/TM-β-CD KP and its displacement to a lower temperature in the curve of RSP/DM-β-CD KP together with the displacement of the RSP exothermic event to different temperatures in both RSP/DM-β-CD KP and RSP/TM-β-CD KP suggest an interaction between drug substance and the two cyclodextrins, as a result of formation of guest–host inclusion complexes. 

### 2.5. FTIR Spectroscopy

**Figure 7:**
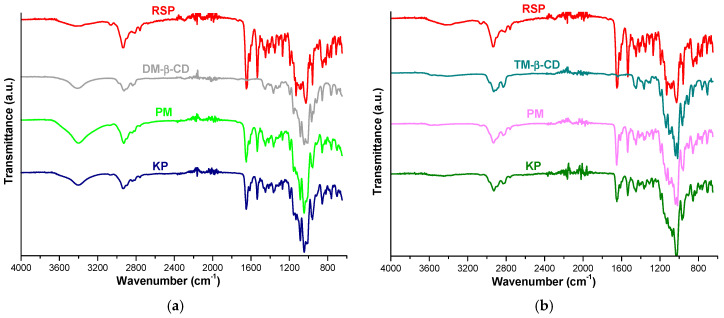
FTIR spectra of RSP, DM-β-CD and their corresponding products PM and KP (**a**); RSP, TM-β-CD and their PM and KP (**b**).

FTIR spectra of RSP, DM-β-CD, TM-β-CD, RSP/CD binary products (PM and KP) are shown in Figure 7. 

The spectral pattern of RSP exhibits characteristic prominent bands at 1648 cm^−1^ assigned to C=O stretching vibration from tetrahydropyrido-pyrimidinone ring, at 1534 cm^−1^ due to C=C stretching vibration of the aromatic ring, at 1027 cm^−1^ corresponding to C_ar_–O stretching from the benzoxazole ring. Other RSP characteristic bands can be noticed at 2936, 2812, 2759 cm^−1^ (aliphatic C–H stretching), at 1414 cm^−1^ (aliphatic C–H bending), at 3063 cm^−1^ (aromatic C–H stretching), at 959, 854, 816 cm^−1^ (aromatic C–H bending). C–N stretching vibration appears at 1352 cm^−1^, C=N stretching arises at 1449 cm^−1^ and C_ar_–F stretching vibration is observed at 1130 cm^−1^ [22,24]. 

The FTIR spectrum of DM-β-CD reveals a broad absorption band in the spectral region of 3500–3300 cm^−1^ assigned to O-H stretching vibration of non-methylated hydroxyl moieties and a large region below 1500 cm^−1^ which displays distinct peaks, characteristic to the cyclodextrin ring [11,36,39]. 

The FTIR spectra of both RSP/DM-β-CD KP and PM show differences in relation with those of the pure compounds. Thus, the RSP bands that correspond to C=C stretching from aromatic ring and C=O stretching vibration shifted from 1534 and 1648 cm^−1^ in pure RSP to 1535 and 1649 cm^−1^ in both PM and KP. In addition, the spectral band associated with aromatic C-H banding from 959 cm^−1^ in pure drug is displaced to 957 cm^−1^ in PM. Furthermore, the characteristic bands of RSP assigned to C_ar_–F stretching, to C_ar_–O stretching and to aromatic C–H bending from 816 cm^−1^ disappeared in binary products KP and PM, giving evidence about the interaction between drug substance and CD (Figure 7a).

The UATR-FTIR spectrum of TM-β-CD shows the characteristic peaks of CD at 2929 cm^−1^ corresponding to symmetric and asymmetric C–H stretching vibration from CH_2_, at 1365 cm^−1^ assigned to C-H bending from CH_2_, at 1016 cm^−1^ due to C–C–O stretching vibration. Moreover, characteristic bands are identified in the spectral region 1076–1022 cm^−1^ attributed to C–O–C stretching vibrations [10,40]. The broad band placed in 3500–3300 cm^−1^ region (O–H stretching vibration), characteristic for DM-β-CD was not observed in the TM-β-CD spectrum due to the methylation of the OH moieties.

Differences are also noticed in the IR spectra of RSP/TM-β-CD KP and PM when compared with spectra of the parent compounds. The RSP characteristic bands associated to C=C stretching vibration of the aromatic ring and C=O stretching vibration located at 1534 and 1648 cm^−1^ in pure RSP spectrum shifted to higher wavenumbers, 1536 cm^−1^ in PM, 1535 cm^−1^ in KP, 1650 cm^−1^ in PM and 1649 cm^−1^ in KP, respectively. Moreover, the RSP band assigned to C_ar_-–O stretching vibration from 1027 cm^−1^ in pure drug is shifted to 1030 cm^−1^ in the PM spectrum and to 1032 cm^−1^ in the KP. The RSP bands that correspond to aromatic C–H bending from 959, 854, 816 cm^−1^ shifted to 957, 856, 825 cm^−1^ in the KP spectrum, and the band associated to aliphatic C–H stretching from 2812 cm^−1^ is displaced to 2816 cm^−1^. Additionally, the band of C_ar_–F stretching vibration from 1130 cm^−1^ in the spectral pattern of pure drug sifted to 1128 cm^−1^ in PM spectrum and it is no more present in the KP spectrum. By analyzing the spectral data, we also found a new band at 1073 cm^−1^ in the KP spectrum without a correspondent in the spectral profile of the parent substances (Figure 7b).

The results of UATR-FTIR studies reveal both the reduction in intensity of RSP bands along with their displacement to different wavenumbers and the disappearance of several characteristic peaks in the KP and PM spectra. These results clearly prove the existence of an interaction between RSP and the two methylated CD and also support the results of molecular docking studies. 

### 2.6. Solubility Profile of RSP/CD Kneaded Products

**Figure 8:**
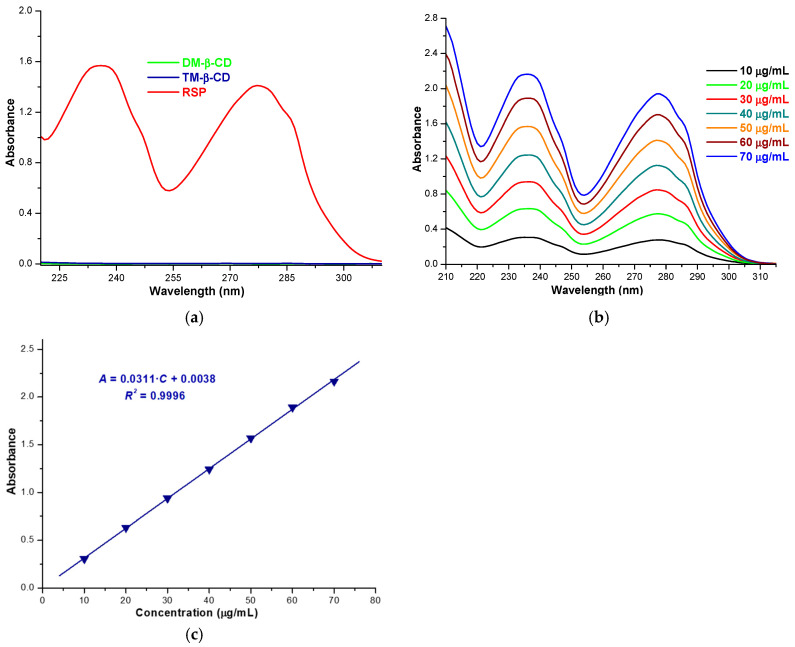
Absorption spectra of: DM-β-CD 130 µg/mL, TM-β-CD 142 µg/mL, RSP 30 µg/mL in phosphate buffer 0.1 M (pH 7.4), at 25 °C (**a**); RSP 10–70 µg/mL in phosphate buffer 0.1 M (pH 7.4), at 25 °C (**b**); RSP calibration curve (**c**).

RSP solubility in the inclusion complexes obtained using the kneading method was assessed by means of the shake-flask method [10,11,41]. The RSP concentration in the saturated solution was evaluated using UV spectrophotometric measurements. Since both DM-β-CD and TM-β-CD solutions in phosphate buffer 0.1 M (pH 7.4) do not present UV absorption in the spectral range of 210–310 nm (Figure 8a), a calibration curve of RSP obtained using absorbance values from 236 nm, at 25 °C (Figure 8b,c) was used to estimate the drug substance in the inclusion complexes. 

The solubility of RSP in the KP with DM-β-CD and TM-β-CD, calculated as an average of five experimental determinations is 1252.063 ± 0.021 µg/mL and 629.627 ± 0.014 µg/mL, respectively. Clear solutions were obtained in standard controlled experiments, when 16.41 mg of RSP/DM-β-CD KP and 8.64 mg of RSP/TM-β-CD KP were dissolved in 5 mL 0.1 M phosphate buffer (pH 7.4) at room temperature.

The saturation solubility studies reveal an increase in RSP solubility in phosphate buffer 0.1 M, pH 7.4 of 1.16-fold in the presence of TM-β-CD and of 2.32-fold in the presence of DM-β-CD as compared with free RSP (540.007 ± 0.003 µg/mL). These results demonstrate the solubilizing effect of the two CD derivatives.

## 3. Materials and Methods

### 3.1. Materials

Risperidone (as Pharmaceutical Secondary Standard) was purchased from Sigma-Aldrich. The two cyclodextrin derivatives, heptakis(2,6-di-O-methyl)-β-cyclodextrin and heptakis(2,3,6-tri-O-methyl)-β-cyclodextrin were acquired from Cyclolab R&L Ltd. (Budapest, Hungary). All other reagents and chemicals used were of analytical purity.

### 3.2. Stoichiometry Determination of RSP/CD Inclusion Complexes 

All UV spectra were recorded using a SPECTRONIC UNICAM—UV 300 UV-VISIBLE double beam spectrophotometer with 1 cm matched quartz cells.

Job’s method was employed to evaluate the stoichiometry of the inclusion complexes between RSP and CDs [10,29]. Job’s plot was generated based on UV spectroscopic measurements. To this end, equimolar 4.87 × 10^−5^ M solutions of RSP and each CD (DM-β-CD and TM-β-CD, respectively) were prepared in 0.1 M phosphate buffer of pH 7.4 and then mixed to a standard volume, varying the RSP molar ratio from 0.0 to 1.0. A dilution set of the RSP stock solution was also prepared similarly, in the same solvent. The solutions were stirred, and the absorbance of all solutions was recorded at 236 nm. The plot of Δ*A* (Δ*A* = *A* − *A*_0_), the difference in RSP absorbance in the presence (*A*) and in the absence (*A*_0_) of CDs vs. RSP mole fraction *R* was generated.

### 3.3. Molecular Modeling Studies

In order to visualize the interaction between RSP, DM-β-CD and TM-β-CD, the molecular docking technique was used. The DM-β-CD structure used in this work was generated from the curated coordinates of ligand 2QKH (X-ray diffraction, resolution 1.9 A) downloaded from the Protein Data Bank database [42]. The methyl groups were manually added on free hydroxyl groups from the 2 and 6 positions (GaussView 5, Semichem Inc., Shawnee Mission, KS, USA) from β-CD. For TM-β-CD the same initial β-CD was used, methyl groups being manually added on all free hydroxyl groups in order to obtain a fully permethylated structure (GaussView 5, Semichem Inc). CDs were optimized in the same manner with RSP (DFT/B3LYP/6-311G). All dihedral angles of the methoxy groups were homogenized, the resulting conformations being compatible with an unhindered CD cavity. Three-dimensional coordinates of RSP were generated using the Gaussian program suite at DFT/B3LYP/6-311G optimization.

As the expected stoichiometry of RSP/CD inclusion complexes was 2:1, two consecutive docking cycles were carried out starting and selecting the best result from each cycle.

The molecular docking analysis was performed using the Autodock 4.2.6 software together with the AutoDockTools [30]. The docking between RSP and CD involves adding all the polar hydrogens, computing the Gasteiger charge; the grid box was created using Autogrid 4 with 50 × 50 × 50 Å in x, y and z directions with 0.375 Å spacing from the CD center. All the calculations were performed in a vacuum. The Lamarckian genetic algorithm with a population size of 150 and a number of 50 runs was applied in the docking process. All the other parameters were used with the default values. Molecular modeling figures were generated using PyMol (The PyMOL Molecular Graphics System, Version 2.0 Schrödinger, LLC, New York, NY, USA) [32]. To validate the docking method’s repeatability and reproducibility we realized redocking and then expressed the results as RMSD in Å using Discovery Studio software. We performed all the calculations in triplicate and expressed them as an average.

### 3.4. Preparation of the Solid Inclusion Complexes and Physical Mixtures

The inclusion complexes of RSP with DM-β-CD and TM-β-CD were prepared using the kneading method in a 2:1 guest:host molar ratio. For RSP/DM-β-CD KP preparation 0.1908 g RSP and 0.3093 g CD were weighed and their mixture was pulverized in an agate mortar and triturated with 0.5 g ethanol:HCl 0.1 M solution (1:1, m/m). To obtain the RSP/TM-β-CD inclusion complex 0.1824 g RSP and 0.3174 g TM-β-CD were accurately weighed and the resulted mixture was pulverized and triturated with 0.50 g ethanol: HCl 0.1 M solution (1:1, m/m) until a homogeneous paste was obtained. The paste was kneaded for 45 min and during this process an appropriate quantity of solvent was added to maintain the paste consistency. The final product was dried at room temperature and then in the oven, at 40 °C for 24 h. The obtained kneaded products were pulverized and passed through a 75-µm size sieve.

Additionally, physical mixtures of RSP and every cyclodextrin in the same molar ratio as the kneaded products were prepared. Risperidone and the host substances were mixed in the agate mortar and pestle for 10 min, in solvent-free manner. 

### 3.5. X-ray Diffraction Studies

The PXRD studies were performed using a diffractometer produced by Bruker, model D8 Advance powder X-ray. The X-ray diffraction patterns were collected with CuKα radiation (40 kV, 40 mA) and a Ni filter over the interval of 5–40° angular domain (2θ). 

### 3.6. Thermal Metods

The thermal profile of RSP, CDs, physical mixtures and kneaded products was evaluated using a Perkin-Elmer DIAMOND TG/DTA instrument. For this purpose samples of about 3–4 mg were placed in aluminum crucibles and were analyzed under air atmosphere at a flow rate of 100 mL min^−1^, over the temperature range of 40–500 °C, with heating rate of 10 °C min^−1^.

### 3.7. FTIR Spectroscopy

The FTIR spectra of RSP, DM-β-CD, TM-β-CD, RSP/CD kneaded products and physical mixtures were recorded using a Perkin Elmer SPECTRUM 100 device. The data were collected directly on solid samples using a UATR device in the spectral domain 4000–600 cm^−1^. Spectra were designed after 16 co-added scans, with a spectral resolution of 4 cm^−1^.

### 3.8. Solubility Profile of RSP/CD Kneaded Products

The saturation shake-flask method was applied to evaluate the change in RSP solubility upon complexation with CDs. An excess amount of RSP, RSP/DM-β-CD and RSP/TM-β-CD kneaded products was added in 5 mL of phosphate buffer 0.1 M (pH 7.4) so that saturated solutions were obtained. After shaking for 24 h at the ambient temperature, the samples were filtered using 0.45 μm cellulose acetate filter. After appropriate dilution, the absorption intensity of the filtrate was recorded at 236 nm. The RSP quantification was carried out by means of calibration curve. To obtain the standard curve a set of RSP solutions in phosphate buffer 0.1 M of pH 7.4 with concentrations between 10 and 70 μg/mL were prepared. The absorbance of the calibration standards was recorded at 236 nm and the data were used to draw the standard curve plotting the absorbance (*A*) against the concentration (*C*, μg/mL). The RSP calibration curve is described by the equation: *A* = 0.0311·*C* − 0.0038, with *R* = 0.9998.

## 4. Conclusions

In this study the molecular encapsulation of antipsychotic drug RSP by two methylated β-cyclodextrin, namely DM-β-CD and TM-β-CD was investigated in solution and in solid state by means of UV-spectroscopy, thermogravimetry, derivative thermogravimetry, heat flow, powder X-ray diffractometry, FTIR spectroscopy and also, by docking studies. The experimental results reveal different physicochemical properties of the binary products obtained by the kneading method as compared to the parent substances, thus suggesting the existence of a real interaction between RSP and CD derivatives as a result of the inclusion complex formation in the stoichiometry of 2:1 as the Job method indicates. The solubility studies highlight a higher increase in RSP solubility when the DM-β-CD is used. The results of this study reveal that the RSP/DM-β-CD inclusion complex can represent a starting point for further research in developing new formulations containing RSP, with enhanced bioavailability and stability.
